# Supplementary material for: Pseudo-colouring an ECG enables lay people to detect QT-interval prolongation regardless of heart rate
Source: PLoS One. 2020 Aug 27;15(8):e0237854. doi: 10.1371/journal.pone.0237854 (PMC7451551; doi:10.1371/journal.pone.0237854)
Supplement: Attachment — Submitted filename: Response to Reviewers.pdf [file pone.0237854.s001.pdf]

Thanks to the reviewers for their comments and suggestions. Below we outline how we addressed each point raised by the reviewers.

Reviewer 1

1- *Please improve the quality of the figures from Fig.4*
We have improved the quality of all figures and double-checked them using the PACE tool provided by PLoS. We also uploaded all full size stimuli used in the study to our GitHub repository, which is referenced in the paper.

2- *The authors are encouraged to provide the details of the multi-reader multi-case Roc and eye-tracking method.*
We have now explained the methods in more detail in the 'experimental design' subsection.

3- *Why authors not followed popular R-peak detection methods? Because the proposed scheme accuracy depends on the correct annotations of ECG events (P-QRS-T and QT interval).*
The pseudo-colouring visualisation technique proposed in the paper does not require any prior annotations of ECG waveforms (P-QRS-T and QT interval); it only requires R-peak detection. As the ECG signals acquired from the clinical trial study were high quality, our R-peak detection method was accurate and efficient. We agree that with low quality ECG signals, using other R-peak detection methods that can reliably distinguish R-peaks from high amplitude noises is essential. We now added this clarification and suggestion under the 'Identifying individual heartbeats' subsection.

4- *In the case of true negative cases, no-coloring schemes are giving better results, whereas, in the case of true positives, the scenario becomes opposite, colored schemes are outperforming. The authors are suggested to add this justification in the discussion section.*
We now discussed these findings in more detail in the 'discussion' section, showing why the specificity (true negative cases) decreased with pseudo-colouring, which was mainly at the QT-level three/borderline to the nomogram.

Reviewer 2

1- *Please change the title too lengthy and not clear. Make a crisp title.*
We have now shortened the title
2- *It is just study or real contribution is there?*
To the best of our knowledge, no prior studies have examined how and to what extent using pseudo-colouring technique can support ECG interpretation. This statement is now added in the 'background and significance' section.
3- *The motivation behind pseudo color any such approaches in literature, needs a clear explanation.*
We have highlighted the part in the 'background and significance' section that explained the motivation behind the pseudo-colouring technique and provided examples of its applications in the literature.
